# Outputs and cost of HIV prevention programmes for truck drivers in Andhra Pradesh, India

**DOI:** 10.1186/1472-6963-9-82

**Published:** 2009-05-21

**Authors:** SG Prem Kumar, Rakhi Dandona, John A Schneider, YK Ramesh, Lalit Dandona

**Affiliations:** 1 George Institute for International Health – India, Hyderabad, India; 2 Health Studies Area, Administrative Staff College of India, Hyderabad, India; 3 School of Public Health and George Institute for International Health, University of Sydney, Sydney, Australia; 4 Department of Medicine, University of Chicago, Chicago, Illinois, USA; 5 Institute for Health Metrics and Evaluation, University of Washington, Seattle, Washington, USA

## Abstract

**Background:**

HIV prevention programmes for truck drivers form part of the HIV control efforts, but systematic data on the outputs and cost of providing such services in India are not readily available for further planning and use of resources.

**Methods:**

Detailed cost and output data were collected from written records and interviews for 2005–2006 fiscal year using standardized methods at six sampled HIV prevention programmes for truck drivers in the Indian state of Andhra Pradesh. The total economic cost for these programmes was computed and the relation of unit cost of services per truck driver with programme scale was assessed using regression analysis.

**Results:**

A total of 120,436 truck drivers were provided services by the six programmes of which 55.9% were long distance truck drivers. The annual economic cost of providing services to a truck driver varied between programmes from US$ 1.52 to 4.56 (mean US$ 2.49). There was an inverse relation between unit economic cost of serving a truck driver and scale of the programme (R^2 ^= 0.63; p = 0.061). The variation between programmes in the average number of contacts made by the programme staff with truck drivers was 1.3 times versus 5.8 times for contacts by peer educators. Only 1.7% of the truck drivers were referred by the programmes for counseling and HIV testing.

**Conclusion:**

These data provide information for further planning of HIV prevention programmes for truck drivers and estimating the resources needed for such programmes. The findings suggest the need to strengthen the role of peer educators and increase referral of truck drivers for HIV testing.

## Background

India has one of the highest numbers of HIV infected persons in the world, estimated at approximately 2.5 million in 2007 [1-3]. The southern Indian state of Andhra Pradesh with a population of 83 million in 2008 is estimated to have the highest HIV burden among the states of India [2].

Truck drivers and cleaners are known to be at increased risk for HIV and other STIs [4]. HIV prevention services for truck drivers in Andhra Pradesh are provided by the Andhra Pradesh State AIDS Control Society (APSACS) through non-governmental organizations (NGOs). With increased availability of public funding for HIV control [5], the prevention activities for HIV are anticipated to increase substantially in the new phase of India's National AIDS Control Programme 2007–2012. Some data on the outputs, cost and efficiency of the various interventions to control HIV in India have become available recently [6-12]. We have recently also reported a composite economic analysis of all public-funded HIV prevention interventions in Andhra Pradesh [13]. We now report details of the outputs, cost and efficiency of publicly-funded HIV prevention programmes for truck drivers in Andhra Pradesh to inform further development of this intervention.

## Methods

The methods used in this study were adapted from our previous studies on cost and efficiency of HIV prevention interventions in the state of Andhra Pradesh [6,7,9,11,12]. This report is part of a study on the cost and outputs of HIV prevention programmes that was approved by the Institutional Ethics Committee of the Administrative Staff college of India, Hyderabad, India.

### Selection of truck driver HIV prevention programmes

At the time of initiating data collection for this study in 2006, 21 HIV prevention programmes for truck drivers were being run by NGOs in Andhra Pradesh with support from APSACS. Six programmes were randomly sampled to obtain a representative sample for the state. The target group covered by these programmes includes both truck drivers and their assistants commonly referred to as 'helpers' or 'cleaners'. In this paper truck drivers and their assistants are referred to as truck drivers.

### Data collection

Data were collected by investigators who received training to ensure a standardized approach to data collection. Standard data collection instruments were used and a pilot study was completed to make final adjustments to the data collection formats and approach. Data were collected from the written records of the programmes and through semi-structured interviews of NGO staff. This included the programme project director, project manager, accounts officer, sexual health service provider, outreach workers and counselors. Formal consent of the senior-most person responsible for each programme, generally the director of non-governmental organisation, was obtained for data collection. Data included a history of the evolution of the programme, and output and cost measurements for each month for the April 2005 – March 2006 fiscal year.

### Outputs data

The total number of individual truck drivers served and the total number of contacts made with them by each programme was documented. According to the programme personnel, a long distance truck driver was defined as a driver or a cleaner who was away from home for more than four continuous days due to work related travel. The National AIDS Control Organisation categorises the services delivered to truck drivers by the HIV prevention programmes into four major components: behaviour change communication (BCC), STI care, condom promotion, and creating an enabling environment [14]. Information on each of the above services was extracted from the written records. BCC comprises several types of sessions by outreach workers, counselors, sexual health service providers and peer educators to teach and encourage the truck drivers individually and in groups to follow safe sex practices. STI care includes the programme staff identifying syndromic manifestations of disease, subsequent referrals for truck driver treatment that could not be completed on site and for HIV counselling at the nearest counselling and testing center. Additional formal training programmes for new sexual health service providers such as registered and private medical practitioners are also included under the sexual transmitted infection care category. Condom promotion includes free condom distribution and sale of condoms under social marketing. Creating an enabling environment for truck drivers includes sensitization meetings with a variety of external stakeholders. These stakeholders include family members of truck drivers, police, media, truck owners associations and policy makers. These meetings assist in establishing linkages with relevant governmental and non-government organisations which includes referral of HIV positive truck drivers to care and support centers and assistance with some of truck driver non-sexual health needs.

### Cost data

Cost data were collected under five categories: salaries, recurrent goods, recurrent services, rentals and capital goods. Economic cost for implementing the HIV prevention programmes was computed rather than just the financial cost, the former being the true resource cost incurred. Similar costing methods were used for all programmes. Indian Rupees (INR) costs were converted into US$ using the average exchange rate of INR 44.27 for a US$ for the April 2005 – March 2006 fiscal year [15].

The salary costs were recorded for all personnel contributing towards the truck driver programmes. Personnel included the project director, project manager, accounts officer, outreach workers, counselor, and attender. Cash compensation paid by the programme to peer educators was also included in salary costs.

Recurrent goods included condoms, medications for STI, BCC materials, stationery and condom outlet boxes. The majority of condoms distributed to the truck drivers through these programmes were provided free of cost by APSACS. The market price of condoms for free distribution is subsidized by up to 70% by the government. We calculated the economic cost of condoms at the unsubsidized cost. Several brands of condoms are also sold to truck drivers through the condom social marketing programmes at the cost at which these are procured. As these condoms are not subsidized, market price was used for the economic cost calculations. The cost of BCC materials was obtained from APSACS which supplies these to the truck driver programmes.

Recurrent services included expenditures for peer educator meetings, training of peer educators and staff, and programme operational costs which included local travel, organization of awareness programmes, mobile clinic set-up cost, organising special events, office maintenance and various other miscellaneous costs. The cost of training was obtained from APSACS. The cost for staff training was calculated by including travel fare, per diem, trainer fees, training materials, and training facility cost.

The offices of all these programmes run by NGOs were located in rented buildings. The details of the monthly rent paid were obtained from the programme records.

Capital goods included computer and accessories, office furniture, electrical fixtures, telephone, audio visual equipment, television, DVD player, public address system, two-wheeled vehicles and air cooler. An attempt was made to obtain the costs incurred on these from the truck driver programme records. When this not available from the programme, the retail market price was determined for these goods. Three quotations for these goods were obtained from the market for the 2005–2006 fiscal year and the average of these was considered as the cost. The life of the capital goods was assumed to be five years, and therefore, one-fifth of the cost was allocated to the 2005–2006 fiscal year if the good was used for the full year. If a capital good was purchased in the middle of this fiscal year and used only for half the year, the cost allocated for this item was half of the yearly cost.

### Data analysis

Data were entered in Microsoft word and excel software, and SPSS version 15.0 was used for data analysis. The average number of contacts made with truck drivers by the programme staff was calculated. The HIV preventions services provided in each of the four HIV prevention service components, i.e. BCC, STI care, condom promotion, and creating an enabling environment, were computed. The number of contacts made with truck drivers by programme staff versus those made by peer educators was compared. The total economic cost for implementing each programme was calculated. The average economic cost per truck driver served in the fiscal year was taken as the main measure of cost-efficiency, and its relation with the total number of truck drivers served assessed using regression analysis. Additionally, the cost per contact with truck driver and its relation with the total number of truck drivers contacted was assessed using regression analysis. The curve estimation regression models available in SPSS (exponential, linear, logarithmic, polynomial, quadratic, cubic and power) were used and the regression forms which yielded the highest R-square values are presented.

## Results

During the 2005–2006 fiscal year, a total of 120,436 individual truck drivers were provided HIV prevention services by the six sampled programmes. The number of truck drivers provided services annually by each programme ranged from 10,824 to 33,127 (mean 20,073). Long distance truck drivers accounted for 55.9% of all truck drivers served (Table 1). A total of 269,476 contacts were made with these individual truck drivers in a year by the six programmes. Programme staff other than peer educators made 56% contacts as compared with 44% made by peer educators. For the six programmes combined, there were on an average 2.46 annual contacts made with each truck driver of which 1.36 contacts were made by programme staff other than peer educators and 1.10 contacts by peer educators. The number of full time equivalents of peer educators in the six programmes had a wider range (14.5 to 44.5, mean 25) as compared with the range for the programme staff other than peer educators (8.3 to 12.3, mean 7.8). The variation in the contacts made by the programme staff with truck drivers was 1.3 times versus a 5.8 times variations in the contacts made by peer educators (Table 1). One programme that had a higher proportion of contacts made annually by peer educators (2.4 contacts per truck driver) as compared with contacts made by programme staff other than peer educators (1.6 contacts per truck driver), had the highest number of BCC and STI related contacts per truck driver annually (3.74 and 0.25, respectively). This programme also had the highest number of condoms distributed per truck driver served (46 annually). Interestingly this programme had only 16 FTEs of peer educators (FTE range for six programmes 15 – 45). We also observed that this programme had the highest unit economic cost per truck driver served (INR. 201.8), and served the least number of truck drivers (10,824) in a year. However, it is important to note that for the same programme, the unit economic cost per truck driver contacted was lower at INR. 50.9 (range INR. 34.6 to INR. 73.1) as the contacts made with each truck driver was high (4 annual contacts).

**Table 1:** Truck drivers served by the six sampled HIV prevention programmes for truck drivers in Andhra Pradesh for 2005–2006 fiscal year.

**Service variable**	**Combined mean**	**Range**
Total truck drivers served	20,073	10,824 – 33,127

*Long distance truck drivers*	11,220	1,297 – 21,993

*Short distance truck drivers*	8,852	260 – 19,472

Total contacts with truck drivers	44,913	20,455 – 64,523

*Contacts by programme staff other than peer educators*	25,295	15,840 – 35,407

*Contacts by peer educators*	19,618	4,615 – 29,777

Contacts with each truck driver by all programme staff combined	2.46	1.56 – 3.97

*Contacts by programme staff other than peer educators*	1.37	1.01 – 2.28

*Contacts by peer educator*	1.10	0.35 – 2.39

Full time equivalent of staff other than peer educators	8	6 – 10

Full time equivalent of peer educators	25	15 – 45

Table 2 shows the intensity of service provision by the six programmes for the various components of HIV prevention services (Table 2). The major portion of contacts with truck drivers by all programme staff was related to BCC (88.6%) with an average of 2.1 contacts with each truck driver. The average annual number of STI care related contacts with each truck driver was 0.16 and the average number of condoms distributed annually to each truck driver was 20. Of all the condoms distributed, 96.8% were provided free by the programmes. Of all the tuckers who received services by these six programmes, 1.7% were referred for counselling and HIV testing (range 0.3–3.4%). The average number of contacts with external stakeholders to create an enabling environment per truck driver served was 0.11, and 91.9% of these contacts were with the community at large in the form of mass and special events and 8.1% with other external stakeholders for advocacy and with agencies to enhance linkages.

**Table 2:** Intensity of services provided by the six sampled HIV prevention programmes for truck drivers in Andhra Pradesh for 2005–2006 fiscal year.

**Component of HIV prevention service**	**Combined mean**	**Range**
Number of contacts related to BCC	42,061	19,445 – 59,087

BCC contacts per truck driver	2.10	1.48 – 3.74

Number of STI care contacts	3,165	1,042 – 6,487

STI care contacts per truck driver	0.16	0.08 – 0.25

Number of condoms distributed	392,930	302,412 – 569,083

Condoms distributed per truck driver	20	10 – 46

Number of contacts relating to creating enabling environment	2,268	270 – 5,683

Creating enabling environment contacts per truck driver	0.11	0.02 – 0.17

BCC is behaviour change communication

STI is sexually transmitted infection

The total economic cost for providing services for the six programmes combined during the 2005–2006 fiscal year was INR 13,284,520 (US$ 300,080), ranging from INR 1,494,626 (US$ 33,762) to INR 3,573,842 (US$ 80,728) with a mean of INR 2,214,087 (US$ 50,013) for the six programmes. The personnel cost accounted for highest share in the total cost with 43.3%, followed by recurrent goods for 31.9% (of this 72.2% for condoms). Recurrent services cost was 21.3% of the total, rentals was 2.2% and capital goods was 1.3%. The economic cost of providing services to a truck driver annually was INR 110.3 (US$ 2.49) with a range of INR 67.5 (US$ 1.52) to INR 201.8 (US$ 4.56) for the six programmes. The economic cost per truck driver contact was INR 49.3 (US$ 1.11) with a range of INR 34.6 (US$ 0.78) to INR 73.1 (US$ 1.65) (Table 3).

**Table 3:** Economic cost of the six sampled HIV prevention programmes for truck drivers in Andhra Pradesh for 2005–2006 fiscal year.

**Service variable**	**Combined mean**	**Range**
Number of truck drivers served	120,436	10,824 – 33,127

Number of truck drivers contacted	269,476	20,455 – 64,523

Total economic cost	INR 13,284,520 (US$ 300,080)	INR 1,494,626 – 3,573,842 (US$ 33,762 – 80,728)

Personnel cost as % of total	43.3	38.5 – 49.1

Recurrent goods cost as % of total	31.9	27.1 – 39.4

Recurrent services cost as % of total	21.3	14.9 – 24.2

Rental cost as % of total	2.2	1.1 – 3.2

Capital goods cost as % of total	1.3	0.8 – 1.8

Cost per truck driver served	INR 110.3 (US$ 2.49)	INR 67.5 – 201.8 (US$ 1.52 – 4.56)

Cost per truck driver contacted	INR 49.3 (US$ 1.11)	INR 34.6 – 73.1 (US$ 0.78 – 1.65)

INR is Indian Rupee; 1 US$ = INR 44.27 in the 2005–2006 fiscal year.

**Figure 1:**
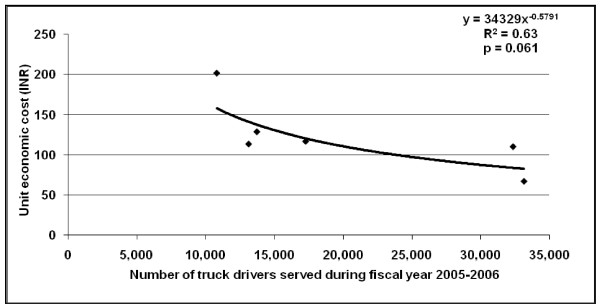
**Relation between unit cost and number of truck drivers served by HIV prevention programmes during the 2005–2006 fiscal year**.

**Figure 2:**
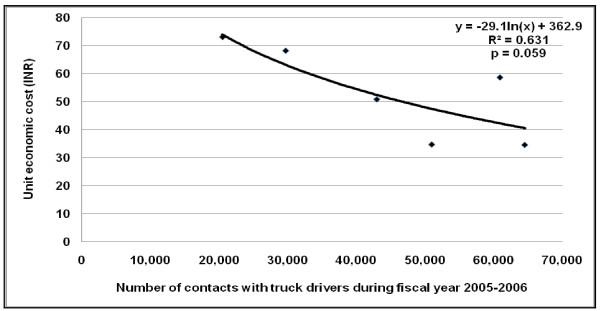
**Relation between unit cost and number of contacts with truck drivers by HIV prevention programmes during the 2005–2006 fiscal year**.

There was an inverse relation between the unit economic cost per truck driver served and the total number of truck drivers served by a programme, with the best fit obtained using a power function (R^2 ^= 0.63; p = 0.061) (Figure 1). A similar inverse relationship was observed between the unit economic cost per truck driver contact and the total number of contacts made with truck drivers by a programme, the best fit for which was obtained using a logarithmic function (R^2 ^= 0.63; p = 0.059) (Figure 2). There was no consistent significant relation between the number of truck drivers served by each programme and the number of contacts with each truck driver in this sample of six programmes.

## Discussion

This analysis of the public-funded HIV prevention programmes for truck drivers in the southern Indian state of Andhra Pradesh revealed that there were on average 2.2 contacts made with each truck driver annually. The annual average economic cost per truck driver served and per truck driver contact was INR 110.3 (US$ 2.49) and INR 49.3 (US$ 1.11) respectively. An important question in resource restricted settings such as India is how best to utilize the limited available resources efficiently that would generate desirable benefits. In a study of HIV prevention in truck drivers in Southern India, an intensive programme consisted of five repeated in-depth interactions with programme staff which demonstrated limited effectiveness [16]. In this context, only 2.2 contacts made annually with each truck driver seem low to yield the desired and sustained intervention impact. The relatively low average number of annual contacts per truck driver served should be interpreted in the background that long-distance truck drivers when on routes outside Andhra Pradesh could be receiving HIV prevention contacts by programmes outside this state [17]. Although data on the number of contacts with programmes outside the state are not available we estimate that it is unlikely that inclusion of these contacts would make the average number of total contacts with long-distance truck drivers several-fold.

The contact by programme staff with truck drivers for behavioural change communication formed a major portion of the work of the programme, constituting 88.6% of the contacts annually. On average 20 condoms were distributed to each truck driver annually by the programmes, although it is possible that some of the long distance truck drivers might have received condoms from programmes outside Andhra Pradesh. Previous reports have suggested a relatively high HIV and STI prevalence and low rates of condom use among truck drivers in India [4,18,19]. We recently estimated that in Andhra Pradesh truck drivers have on average about 100 sex acts annually, of which about half are with paid or other non-regular partners [13]. A recent report on long-distance truck drivers on major national highways in India reported that of the 513 truck drivers using the south-east route passing through Andhra Pradesh, in the last 12 months 223 (43.5%) had sex with paid female partner for which 64.3% of those used condom consistently, 107 (20.8%) had sex with non-paid female sex partner other than spouse for which 14% of those used condom consistently, 6 (1.2%) had sex with a male or transgender for which 16.7% of those used condom consistently, and almost all the 422 married truck drivers had sex with their wife of which 0.5% used condom consistently [20]. Findings from these various sources suggest more work is needed to boost safe sex and condom use by truck drivers in Andhra Pradesh.

Of the truck drivers served by the six programmes, 15.8% were treated for sexually transmitted infections. Only 1.7% of the truck drivers were referred for counseling and HIV testing, a very small proportion. It is also important to note that HIV prevention services were provided by these programmes only during the day time, missing the truck drivers passing through during night. These findings highlight the need to increase referrals for HIV counseling and testing and the need to increase night-time HIV prevention services by these programmes.

Of all the contacts made with truck drivers by for HIV prevention services, about half made by peer educators. There was significant more variation between the programmes in the number of contacts made by peer educators as compared with the contacts made by the regular programme staff. While this variation for the programme staff was 1.3 times it was 5.8 times for peer educators. Peer educators for the truck drivers programmes generally include employees of transport companies, eateries and petrol pumps at halting stations. Special attention to strengthening the role of peer educators is needed because they are more easily accessible to truck drivers, especially at night time, as compared with the regular programme staff. Attention to ensuring quality service provision by peer educators is needed through organization of periodic training programmes and monitoring.

We found that the cost-efficiency improved with increasing number of truck drivers served by the programmes as well as with increasing number of truck drivers contacted by the programmes, but this relation fell slightly short of reaching statistical significance of p < 0.05 probably because of the small sample of programmes in this study. We have previously reported generally similar relation between cost-efficiency and scale for other HIV interventions in Andhra Pradesh [6,7,9,11,12]. However, efforts at increasing efficiency of a programme by increasing scale have to take into consideration that this does not lead to compromise of quality of services provided.

We used output data provided by the programmes for our analysis. Though we made significant efforts to verify the data from written records as far as possible in this cross-sectional study, more accurate data may be possible through prospective studies. Another limitation of our study is that our data do not allow correlating programme outputs with programme impact on behavioral and biological variables, which again would be possible in prospective studies.

## Conclusion

The outputs, cost and efficiency estimates reported in this paper for the truck driver programmes in this Indian state could be useful for further planning of HIV prevention programmes for truck drivers and estimating the resources needed for such programmes. The data presented in this paper emphasize the need for strengthening the role of peer educators and for increasing the referral of truck drivers for counseling and HIV testing. The findings also suggest that increasing the scale of the programmes may lead to better cost-efficiency but caution is needed that this does not lead to worsening of the quality of HIV prevention services provided.

## Competing interests

The authors declare that they have no competing interests.

## Authors' contributions

SGPK contributed to the design, data collection, analysis, interpretation, and wrote the first draft of the paper. RD contributed to the analysis, interpretation and paper writing. YKR contributed to the design, data collection, and analysis. JS contributed to the interpretation and paper writing. LD led the main study from which this report is derived, and contributed to the design, data collection, analysis, interpretation and paper writing. All authors except YKR (deceased) approved the final version of the paper.

## Pre-publication history

The pre-publication history for this paper can be accessed here:


